# The occupational anxiety of teachers caused by China’s ‘double reduction’ policy—a study based on the grounded theory

**DOI:** 10.3389/fpsyg.2023.1144565

**Published:** 2023-03-22

**Authors:** Wei Yue, Le Yu, Yanru Yang

**Affiliations:** School of Education, Central China Normal University, Wuhan, China

**Keywords:** the ‘double reduction’ policy, teachers’ occupational anxiety, the grounded theory, the implementation of policy, actual educational ecology

## Abstract

Teachers’ occupational anxiety is a kind of negative emotional state of teachers, which is prevalent in Chinese teachers. Unfortunately, in the existing research, teachers’ occupational anxiety caused by China’s ‘double reduction’ policy has not been paid attention to. Based on the grounded theory, this study conducted in-depth interviews with 45 in-service primary and junior high school teachers, and used NVivo 12 to process recording materials. Through a series of steps such as open coding, axial coding and selective coding, we found that the core feature of teachers’ occupational anxiety caused by the ‘double reduction’ policy was that the implementation of the ‘double reduction’ policy was incomplete matching the actual educational ecology. Then we constructed a theoretical model of the formation mechanism of teachers’ occupational anxiety caused by the ‘double reduction’ policy. The study showed that due to the influence of teachers’ own personality characteristics and incomplete match between external factors, although teachers insisted on self-adjustment, it was difficult to fundamentally solve the teachers’ occupational anxiety caused by the ‘double reduction’ policy.

## 1. Introduction

In the 21st century, anxiety has become the central issue of human existence and an important theme in many aspects of modern life (Sinclair and Ryan, 1987). With the continuous development of society, teachers are faced with fast-paced changes in educational practice and expectations, and this rate of change to some extent may contribute to an increase in anxiety (Henderson and Corry, 2021).

### 1.1. Anxiety and teachers’ occupational anxiety

Anxiety is an unpleasant and complex emotional state of tension, uneasiness, worry, and annoyance that an individual experiences in response to an impending and potentially dangerous or threatening situation. Spielberger (1966) made the important distinction between anxiety as a transitory state (A-State) and as a relatively stable personality trait (A-Trait). He suggested that state anxiety was situational and involved fear following autonomic nervous system activity, whereas trait anxiety was an acquired behavioral tendency. As one of the groups prone to anxiety, occupational anxiety in teachers is a risk factor that develops under the effect of long periods of chronic stress, i.e., reduced job satisfaction, burnout and poor performance due to excessive job stress that affects their daily functioning and emotional balance (Agyapong et al., 2022). At the same time, teachers’ excessive occupational anxiety will also seriously affect students’ motivation and performance, thus causing students’ anxiety, which in turn may maintain or even increase teachers’ anxiety (Sinclair and Ryan, 1987). Therefore, focusing on teachers’ occupational anxiety and exploring strategies to address it has received high attention from relevant research and practice.

### 1.2. Teachers’ occupational anxiety

Regarding teachers’ occupational anxiety, studies had found that the types were mainly related to subject anxiety, such as mathematics (Peker and Ertekin, 2011; Ganley et al., 2019; Bosica, 2022), English (Liu and Wu, 2021), and science (Novak et al., 2022). For example, Bosica (2022) investigated mathematics teaching anxiety and mathematics anxiety among in-service elementary school teachers in Ontario. By collecting data from 185 questionnaires from pre-service elementary school teachers in six teacher education programs in Ontario and interviewing 16 of them, the researchers found that mathematics teaching anxiety and teachers’ mathematics anxiety were both significantly related and interacted with each other. Liu and Wu (2021) found that anxiety affects teachers’ work and life by analyzing data collected from mixed-form questionnaires. Ganley et al. (2019) used Rasch analysis to conclude that science teaching anxiety and science interest were significant predictors of teaching self-efficacy in preservice elementary teachers. However, different from previous studies, this study does not focus on a single discipline to analyze teachers’ occupational anxiety, but intends to study teachers’ occupational anxiety from the perspective of the whole discipline.

It was precisely because of the concern about the widespread existence of teachers’ occupational anxiety that researchers gradually turned their focus to explore the source of teachers’ occupational anxiety. Some studies suggested that teachers’ occupational anxiety was not only related to internal factors such as teachers’ negative experiences or behaviors in the classroom (Bekdemir, 2010), low physical activity (Biernat et al., 2022), teachers’ psychosocial burden (Umiastowska and Gdaniec, 2016; Nazaruk and Marchel, 2019), fear of language and negative results, and lack of confidence in their own abilities (Liu and Wu, 2021), but also affected by external factors such as district (Cheung and Hui, 2011), COVID-19 (Pressley et al., 2021; Alhazmi et al., 2022; Koestner et al., 2022), social and professional conditions (Alasheev and Bykov, 2002), and the incompatible relationships between supervisors and teachers (Youngs, 1978). In addition, some researchers pointed out that teachers’ occupational anxiety was the result of multiple factors, that was, the main source of anxiety was related to time demands, pupils’ difficulties, large class enrollments, financial constraints and lack of educational resources (Coates and Thoresen, 1976). In fact, from a causal perspective, teachers’ occupational anxiety as a negative emotional state, it was not only aggravated teachers’ job burnout (Genoud and Waroux, 2021), but also seriously affected students’ learning results or effectiveness (Stanton, 1974; Youngs, 1978). Therefore, the researchers believed that teachers’ occupational anxiety should be alleviated and the conflict between work and life should be resolved through systematic desensitization and teaching technology guidance (Coates and Thoresen, 1976), periodical medical evaluation of teachers (Desouky and Allam, 2017), and parenting intervention (Haslam et al., 2013). Meanwhile, some researchers pointed out that in the context of the COVID-19, teachers needed to actively communicate with the community and found targeted activities to deal with occupational anxiety caused by the epidemic while complying with quarantine requirements (Talidong and Toquero, 2020).

Although the above research has analyzed teachers’ occupational anxiety from different dimensions, there is no research to explore teachers’ occupational anxiety against the background of national education policies. In the application of research methods, more research has been done using cross-sectional questionnaire surveys, anxiety scales and tests, and less research has been done using qualitative methods to analyze teachers’ occupational anxiety. In addition, previous studies have mostly interpreted the negative impact of teachers’ occupational anxiety on students’ learning, and less and difficult to focus on the negative impact on teachers’ own development. Therefore, this research takes China’s ‘double reduction’ policy as the background, and uses grounded theory to deeply explore the main characteristics, root causes and teachers’ self-adjustment of teachers’ occupational anxiety, in order to further clarify the internal mechanism of teachers’ occupational anxiety, which will be of great significance to how to effectively alleviate teachers’ occupational anxiety and promote teachers’ professional development.

### 1.3. China’s ‘double reduction’ policy

Since ‘double reduction’ is an education policy with Chinese characteristics, it is also extremely necessary to understand the existing research on ‘double reduction’. The so-called ‘double reduction’ means that on July 24, 2021, the General Office of the Central Committee of the Communist Party of China and the General Office of the State Council issued the Opinions on Further to Ease the Burden of Excessive Homework and Off-campus Tutoring for Students undergoing Compulsory Education. In this context, all sectors of society focus on how to effectively reduce the burden on students, how to effectively rectify off-campus training institutions, and continue to observe the effectiveness of ‘double reduction’. Educational researchers have thought about the implementation of the ‘double reduction’ strategy from multiple perspectives, such as innovating after-school service mode, enhancing after-school service guarantee, improving primary and secondary school homework to meet students’ personalized homework needs (Yang, 2021), and improving public education psychology (Zhang et al., 2022). At the same time, some scholars noticed that the excessive burden of schoolwork not only reflected the fatigue of students, but also reflected the anxiety of parents. They proposed to reshape the educational ecology of students’ healthy growth by formulating scientific growth standards, strengthening the cooperative education of family-school-society, and believed that the problem of parents’ group anxiety had become an increasingly serious social problem. Among them, the core of parental anxiety was whether children could achieve upward mobility of social class through education (Zhou and Fu, 2021). In a word, the research on ‘double reduction’ is extremely rich. However, scholars mostly analyze the implementation strategies and effects of ‘double reduction’ from the perspective of students and parents, but ignore the important role and status of ‘teachers’ as ‘double reduction’ participants, which makes the research inevitably have certain limitations. Although some studies also point out that teachers need to ‘teach as much as they can’, grasp the key links and important nodes, build a bridge of knowledge and experience (Ke et al., 2022), and rely on digital education resources to improve the efficiency and quality of the classroom, they all point to teachers’ development as it should be, without considering the actual state of teachers during the implementation of ‘double reduction’, especially the occupational anxiety of teachers. To sum up, whether from the existing research on teachers’ occupational anxiety or the current research progress on China’s ‘double reduction’ policy, it is necessary and innovative to study teachers’ occupational anxiety against the background of the ‘double reduction’ policy.

## 2. Materials and methods

**Figure 1:**
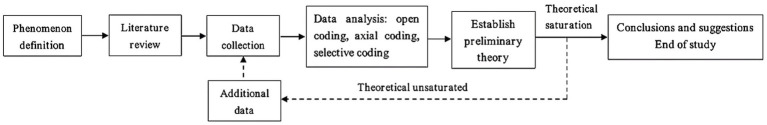
Flow chart of grounded theory.

This study adopts the grounded theory method invented by Glaser and Strauss (1967), aiming to explore teachers’ occupational anxiety caused by the ‘double reduction’ policy and the complex factors behind it, and then generate the corresponding substantive theoretical model. The main steps of the grounded theory include open coding, axial coding and selective coding (the specific process is shown in Figure 1). The grounded theory opposes the deductive paradigm of using experience to yield to theory, and advocates finding theory from experience, and then using theory to reflect experience and serve the understanding of experience. The main goal of the grounded theory is to generate a theory from empirical materials to explain a behavior pattern, which is related to participants or the problems involved by participants (Glaser, 1978).

We use the grounded theory method in qualitative research to systematically collect and analyze data, mainly for the following reasons. First, since the purpose of this research is to explore the internal formation mechanism of teachers’ occupational anxiety caused by the ‘double reduction’ policy, the deductive method does not apply to such research. As a method based on inductive reasoning, the grounded theory is conducive to generating a theory based on data and formed by the views of participants, to really go beyond description and toward a theoretical interpretation of processes or phenomena (Turner and Astin, 2021). Second, the use of grounded theory can further tap teachers’ real ideas about occupational anxiety and some hidden information. At some stages of the theoretical development process, the use of existing theories can not only enlighten research, but also further add new theoretical content to enrich the theoretical basis (Goldkuhl and Cronholm, 2010).

### 2.1. Participants

Since China’s ‘double reduction’ policy was mainly aimed at the compulsory education stage (primary and junior high schools), the researchers recruited 45 qualified in-service primary and junior high school teachers by issuing electronic recruitment leaflets, and took them as the research objects. In order to improve the representativeness of the sample, the researcher also comprehensively considered various factors such as teacher’s gender, length of service as a teacher, region, difference in schooling stages and disciplinary differences during recruitment. Specifically, considering the different length of service as a teacher, their occupational anxiety would also be different. Therefore, the researchers divided the teaching experience of teachers into three growth stages: novice teacher, proficient teacher and expert teacher. The research showed that the teachers with teaching experience of 1–3 years and relatively high level of pre-class teaching strategies, but lack of flexibility in teaching process, belonged to novice teachers, the teachers with teaching experience of 4–14 years and higher level of in-class teaching strategies, and were familiar with the content and procedures related to the class, belonged to proficient teachers, while the teachers with teaching experience of 15 years or more, who had stronger innovation and reflection consciousness than proficient teachers, belonged to expert teachers (Luo, 2006; Dong and Dong, 2022). As many novice teachers just had the education and teaching experience before and after the ‘double reduction’ policy was issued, the teachers’ occupational anxiety caused by the ‘double reduction’ policy was more obvious to them. While proficient teachers were in a critical period of growth, their probability of producing occupational anxiety was far greater than that of expert teachers with relatively complete literacy in all aspects. According to the information received in the electronic recruitment leaflets, the researcher selected a certain number of qualified novice teachers and proficient teachers. At the same time, in order to ensure the rigorous nature of the research, the researcher also selected some expert teachers for interviews. Therefore, the sampling of this study focused more on novice teachers and proficient teachers. At the same time, as far as the region was concerned, it was mainly divided into urban and rural areas. When sampling, the researchers fully considered the comprehensive development level of the region. Therefore, the urban and rural areas selected in this study were regions with moderate development level, which could represent the basic situation of the whole China to a certain extent, and were highly representative. Specific sample information is shown in Table 1.

**Table 1:** Sample distribution (total = 45).

Characteristic	Classification	Numbers	Percentage
Gender	Male	19	42.22%
	Female	26	57.78%
Length of service as a teacher	Novice teacher	11	24.44%
	Proficient teacher	25	55.56%
	Expert teacher	9	20.00%
Region	Urban area	27	60.00%
	Rural area	18	40.00%
Schooling stage	Primary school	24	53.33%
	Junior high school	21	46.67%
Discipline	Chinese	7	15.56%
	Math	7	15.56%
	English	6	13.33%
	Politics	3	6.67%
	History	5	11.11%
	Geography	4	8.89%
	Physics	3	6.67%
	Chemistry	4	8.89%
	Biology	2	4.44%
	Music	2	4.44%
	Art	1	2.22%
	PE	1	2.22%

### 2.2. Procedure

**Figure 2:**
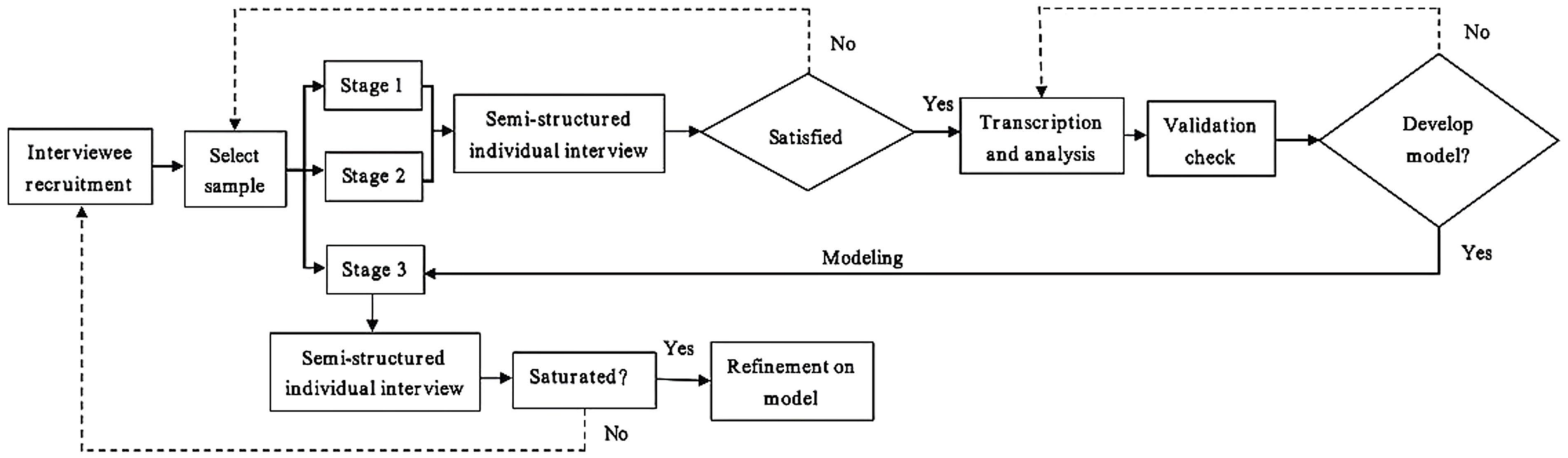
Flow chart of data collection and analysis based on grounded theory.

The grounded theory is not only a methodology, but also a method for exploratory research (Turner and Astin, 2021). There are three basic elements of grounded theory: concept, category and proposition. Among them, concept is the basic unit of analysis, because theory is developed from the conceptualization of data rather than the actual data itself (Pandit, 1996). Therefore, this study conducted data collection in three stages based on the principle of theoretical sampling. Semi-structured interviews were used in each stage, and samples were timely increased according to the actual development of the theory until the categories in the data reached theoretical saturation. In addition, this study always adhered to the ethical and moral principles in education research, fully respected the personal privacy and willingness of the interviewees, and only after obtaining the permission of the interviewees in advance, could the interview be recorded in the whole process. The duration of each interview was between 30 and 60 min. At the same time, when sorting out the data, the names of relevant schools and people were also anonymized (see Figure 2 for details).

#### 2.2.1. Stage 1

This stage was to initially construct the interview system and coding system. First of all, the researchers worked out an interview outline around the theme and purpose of the study (see Table 2). Due to the impact of the COVID-19, the researchers chose to flexibly adopt online interviews. Subsequently, the researcher selected 20 subjects as the preliminary sample, including 11 primary school teachers and 9 junior high school teachers. The subjects involved include Chinese, math, English, politics, history, geography, physics, chemistry, biology, music, art and PE. Based on the proposed interview outline, the researcher conducted one-to-one semi-structured interviews with these in-service teachers. Although the interview outline was designed around the overall logic of the main representations of teachers’ occupational anxiety caused by the ‘double reduction’ policy, the causes of anxiety and the countermeasures to deal with anxiety, in the actual interview process, when researchers found some new key points, they would also ask questions at appropriate times. Finally, at the end of all interviews, three researchers transcribed the interview recordings word for word in time, and further coded and analyzed the interview text.

**Table 2:** Interview outline.

Number	Questions
1	The ‘double reduction’ policy has been implemented for more than a year. How do you think it works?
2	Do you feel anxious in the process of implementing the ‘double reduction’ policy? Why?
3	What do you think is the main source of anxiety?
4	What impact do you think anxiety has on your work, life, body and psychology? How do you adjust yourself?
5	If you were given the choice now, would you still choose the profession of teachers? Please explain the reason.
6	How do you think to deal with the anxiety caused by ‘double reduction’ policy?

#### 2.2.2. Stage 2

In this stage, the coding system developed in the first stage was further confirmed and improved. The researcher chose 15 in-service primary and junior high school teachers as the subjects of this round of research, and still adopted the online one-to-one semi-structured interview method, aiming to enrich the attributes and dimensions of the emerged categories and further explore the underlying reasons behind teachers’ occupational anxiety caused by the ‘double reduction’ policy. After this round of interviews, the researcher continued to code the interview texts in the same way as in the first stage. At this stage, the existing categories were further enriched. The relationships among the categories were also clarified through the paradigmatic model of axial coding. Based on this, the researcher established the core category through selective coding and developed a theoretical model of the formation mechanism of teachers’ occupational anxiety caused by the ‘double reduction’ policy.

#### 2.2.3. Stage 3

This stage was mainly to confirm or revise the theoretical model formed in the second stage. The researcher continued to select 10 subjects for a new round of online one-to-one semi-structured interview. After this round of interviews, the researchers found that there were no new categories or attributes, and the theoretical model of the formation mechanism of teachers’ occupational anxiety caused by the ‘double reduction’ policy formed in the second round was further confirmed here. This meant that the study achieved theoretical saturation so far, and no new data need to be collected.

### 2.3. Data analysis

Coding is a necessary step for empirical data to gradually form a theory. This study was based on the three-level coding strategy of ‘open coding-axial coding-selective coding’ invented by Corbin and Strauss (1990), and analyzed the collected interview data layer by layer with the help of Nvivo12 software.

**Figure 3:**
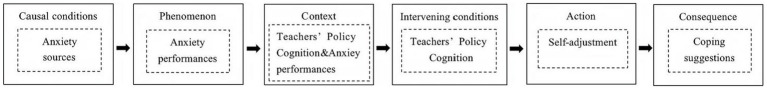
Schematic diagram of the axial coding of teachers’ occupational anxiety.

**Figure 4:**
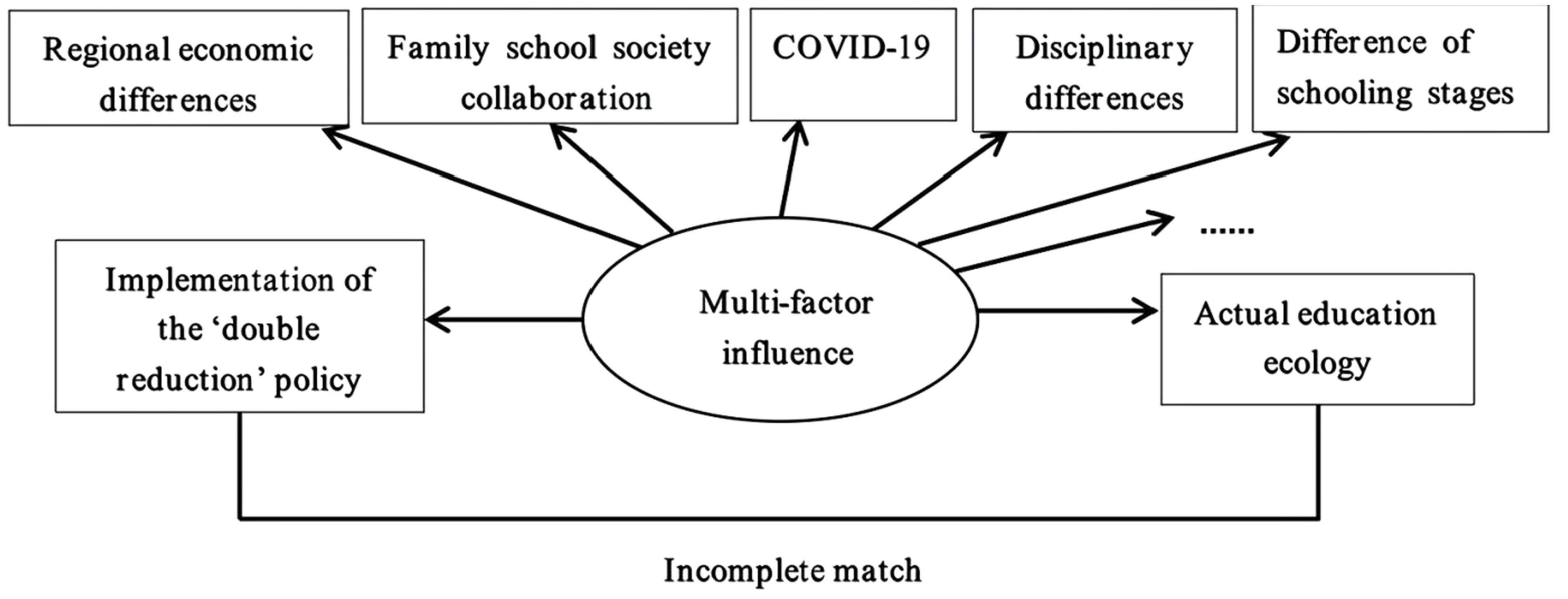
The relationship between the implementation of the ‘double reduction’ policy and actual education ecology.

**Figure 5:**
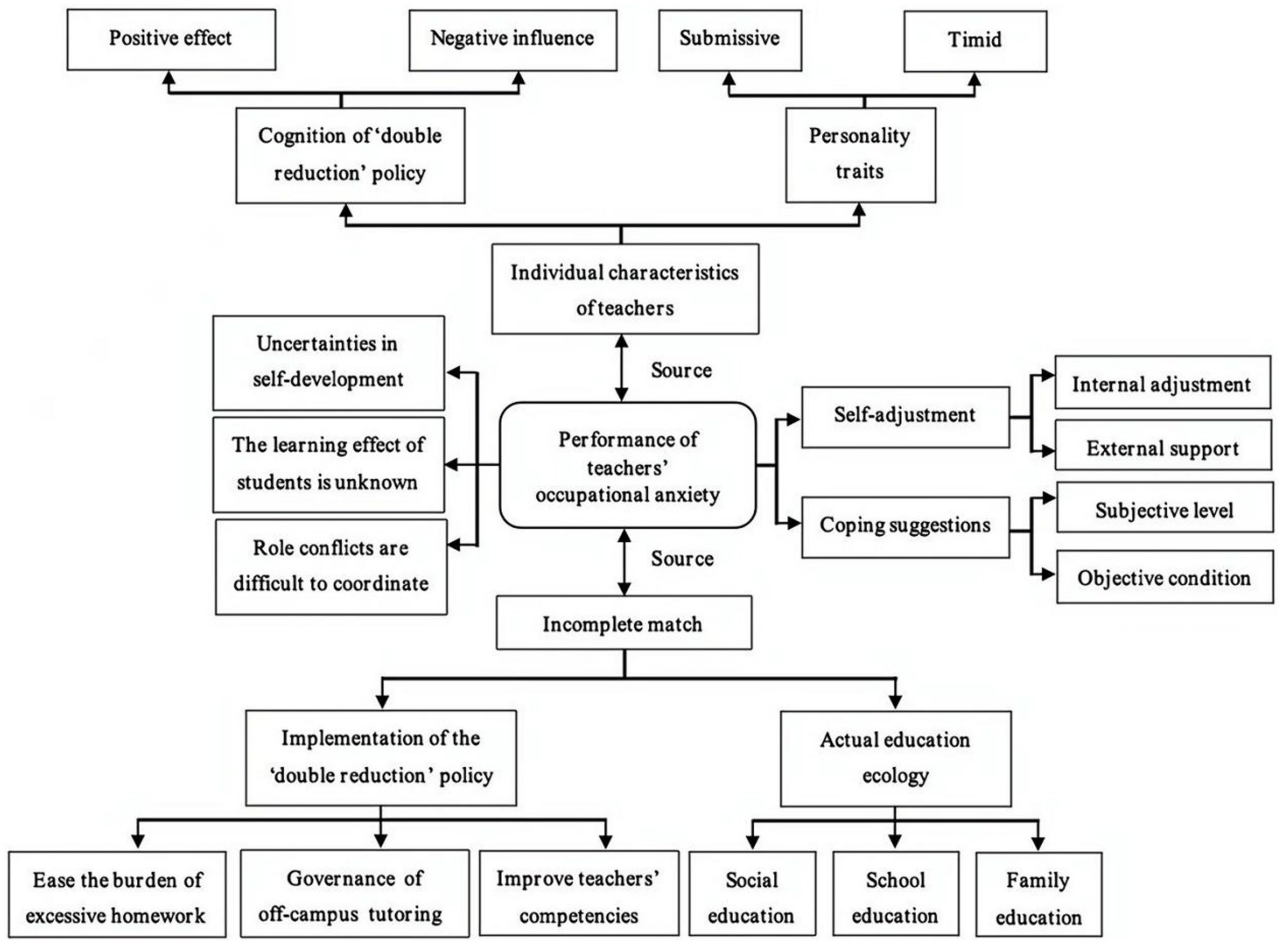
Theoretical model of the formation mechanism of teachers’ occupational anxiety caused by the ‘double reduction’ policy.

The first stage of data collection and analysis was open coding, which aimed to disassemble, compare, label, conceptualize, and categorize the data. For example, the label ‘teacher growth’ was given to ‘the introduction of the policy of double reduction has forced some teachers to change the old and conservative teaching methods, which can force teachers to grow’. As the new label ‘richer curriculum’ emerged in the supplementary interviews, a new correlational concept ‘positive effect’ could be created based on the existing labels, and further categorized into the category ‘teachers’ policy cognition’. Through the use of induction and in-depth comparison, five major categories were created at this stage (see Table 3). For the axial coding, it was primarily located in the second phase of data collection and analysis, which centered on the establishment of a paradigm model and the determination of the relationships among categories. After analyzing the relationship of the five categories, the study clarified the status of ‘teachers’ occupational anxiety’ as a major category (see Figure 3). In addition, the third stage of data collection and analysis was the selective coding, the core of which was to write a story line to construct a theoretical model of the formation mechanism of teachers’ occupational anxiety caused by the ‘double reduction’ policy through the selection of core category (see Table 4; Figures 4, 5).

**Table 3:** Open coding table of teachers’ occupational anxiety caused by the ‘double reduction’ policy.

Original statements (partially listed)	Labeling	Initial categories	Major categories
T2: The introduction of the policy of ‘double reduction’ has forced some teachers to change the old and conservative teaching methods, which can force teachers to grow.	Teacher growth	Positive effect	Teachers’ policy cognition
T37: From the perspective of students, their homework is really less now, and their sleep time and after-school time are relatively increased.	Reduce students’ pressure		
T24: The school will offer different activity curriculums, and we will buy books and teaching aids specially, so that students can have more activities.	Richer curriculum		
T14: The ‘double reduction’ does not reduce the burden of teachers, although on the surface, the amount of homework reduced, the teacher needs to correct the amount of homework will be reduced, but the policy has been implemented, the school’s requirements have never been reduced.	Teachers’ burden	Negative influence	
T4: It only temporarily relieves students’ pressure, but when students reach the third grade, you will find that the gap between the academic levels of children in the class is too large, which is actually just a pseudo behavior of the first and second grades.	Pseudo learning behavior		
T8: In fact, most families do not lower their learning requirements for their children, but still try to improve their children’s performance through tutoring and other ways. Therefore, the pressure on students has not been reduced.	Students’ pressure		
T1: The school attaches great importance to the quality of students, that is, students’ achievements are actually linked to the stability of teachers’ work. Before I came, some teachers were dismissed because they did not achieve positive results.	Post stability	Self-development	Anxiety performances
T16: When I am anxious, I will doubt myself and wonder whether my ability can make these children accept my teaching.	Teaching ability		
T40: It is totally unequal to effort and reward. For example, after 2 months of after-school service last semester, the final reward may be only 200 yuan.	Effort and reward		
T10: In the limited time, if students want to achieve the same or even better results, the efficiency of the classroom must be improved.	Learning effect	Student learning	
T13: I usually go to work very early and go home very late, which does not correspond to the school time of my children. It’s really contradictory. The anxiety of not having time to accompany my children is actually a guilt for them.	Internal of individual family	Role conflict	
T31: To tell the truth, teachers in our grade are very competitive. They sacrifice lunch time to help students.	Teacher colleague		
T20: My anxiety comes from the handling of some conflicts between family and school. I really get a call from parents of students at zero o’clock and swear at you.	Family and school communication		
T43: I think my anxiety is also related to ‘double reduction’, because students really have many ways to acquire knowledge, but now their only approach is to study at school. Therefore, teachers are bound to be anxious under greater pressure.	Approach narrowing	The ‘double reduction’ policy	Anxiety sources
T5: I think the source is our sense of responsibility as teachers. If we want to teach students well, we will bear a lot of pressure and it is easy for us to be anxious.	Inner sense of responsibility	Teachers’ individual factors	
T42: I do not think I have a good time arrangement, or I have not formed the habit of making full use of fragmented time to improve myself, so it is difficult to balance work and life, and eventually I will have anxiety.	Time planning		
T11: The pressure exerted by parents on teachers is also great.	Parents exert pressure	Parents’ weak coordination	
T26: When I feed back my children’s learning to parents, some parents are indifferent and do not cooperate, so I will be anxious.	Incompatibility of parents		
T17: Because I am good at adjusting my mentality, I will not bring anxiety into my life, but also try to turn anxiety into motivation in my work.	Mentality	Internal adjustment	Self-adjustment
T19: Because I need to digest my negative emotions after work, I will eat a lot, which leads to my weight gain of about 10 kg last year.	Diet		
T6: I am very busy from Monday to Friday, and I work overtime for work. Therefore, as long as I have time on Saturday and Sunday, I just want to rest and sleep until I wake up naturally.	Sleep		
T18: I always try to improve my work efficiency, so that when I go home, I will not spend so much time on work.	Reasonable planning		
T33: At home, my mother helps to take care of the children, so it lightens my burden.	Family support	External support	
T21: Our school has a good humanistic environment, and the overall working atmosphere is very comfortable.	Humanistic care of schools		
T15: For example, our lesson preparation group, I think, is a united family, and we will solve problems together.	Collaborate with colleagues		
T28: First of all, as teachers, we should be able to adjust our mentality in time.	Adjust mentality	Subjective level	Coping suggestions
T34: We should improve our work efficiency and allocate tasks reasonably, so that we will not pile things up in 1 day or one node to complete.	Improve work efficiency		
T22: Teachers should have the courage to speak out. When they are under great pressure and facing difficulties in their work, they should communicate with leaders and learn to reduce their responsibilities appropriately.	Appropriate reduction of responsibility		
T3: Teachers should learn more and improve their professional quality.	Persevere in study		
T7: We should recognize the rationality of the ‘double reduction’ policy and actively cooperate in our work.	Actively adapt to policy		
T9: I think ‘double reduction’ really needs to be implemented from top to bottom. Teachers have paid their time and their salaries must be in place.	Improve policy	Objective condition	
T12: What I feel deeply is the mutual support and help between schools, leaders and teachers. It is a very good way to establish a united and cooperative relationship.	Building collaboration		

**Table 4:** Core category and refinement analysis.

Core category	Attribute	Dimension
The incomplete match between the implementation of the ‘double reduction’ policy and the actual educational ecology	The implementation of the ‘double reduction’ policy	Valid-Invalid
	Actual education ecology	Good-Bad

### 2.4. Theoretical saturation test

Using the grounded theory approach, the researcher needs to continuously collect and analyze data and selectively code them until saturation is achieved, resulting in a comprehensive and parsimonious theory based on the data (Urquhart et al., 2010). In order to confirm the possibility of generating new categories and concepts, after completing the first phase, we organized a second phase of data collection and analysis, which indeed revealed the generation of new categories and concepts. Based on this, we organized a third phase of collection and analysis, but no new information was found, indicating that the constructed theoretical model was saturated. At the same time, we fed back the category and model formed after coding to the interviewees, who confirmed that they were fully compatible with the reality and that no new category needed to be added. Given the clarity and robustness of the extracted major category, initial category and relationship descriptions, we then stopped collecting new data.

### 2.5. Rigor

Because qualitative research values the depth of the study and has a more complex research process, for sample size, qualitative research is not like quantitative research that selects a large sample, but rather the sample is generally more appropriately controlled at 30 or less (Burns and Schneider, 2019). This study strictly followed the principles of theoretical sampling to ensure a rich sample size and research data. In addition, the researcher also attached great importance to the continuous reflection on the research process, and after each interview and analysis of phased data, they timely recorded the research content that needed to be further improved or updated, making the research at each stage more rigorous.

### 2.6. Ethical considerations

The need for ethical approval was waived by the university. No personal identifiable information was gathered, such as name, living address, ID, and telephone number. The study adopted one-to-one semi-structured interviews. The duration of each interview was between 30 and 60 min.

## 3. Results and theory

Through theoretical sampling and a rigorous three-level coding procedure, this study clarified the basic representations of teachers’ occupational anxiety induced by the ‘double reduction’ policy, teachers’ objective perceptions of the policy and anxiety, and their personal perceptions of how to cope with anxiety. The responses to these questions formed a solid theoretical foundation for teachers’ occupational anxiety caused by the ‘double reduction’ policy. This part will take the three-level coding as the clue to promote the data analysis layer by layer until the theory comes into being.

### 3.1. Open coding

Through fine coding of all interview texts, in the open coding stage, this study summarized all the data into 12 initial concepts: positive effect, negative influence, self-development, student learning, role conflict, the ‘double reduction’ policy, teachers’ individual factors, parents’ weak coordination, internal adjustment, external support, subjective level and objective condition. After further comparative analysis, five categories were extracted: teachers’ policy cognition, anxiety performances, anxiety sources, self-adjustment and coping suggestions (see Table 3). With regard to the detailed information of these categories and concepts, this study will make a specific description in the subsequent selective coding part.

### 3.2. Axial coding

On the basis of the open coding, according to the paradigm model of Corbin and Strauss (1990), this study recombined the category and attribute of teachers’ occupational anxiety caused by the ‘double reduction’ policy, and distinguished the main category from the sub category by analyzing the causal conditions, context, intervening conditions, action and consequence of the phenomenon.

It can be seen from Figure 3 that teachers’ occupational anxiety caused by the ‘double reduction’ policy is the main category of the study, and teachers’ policy cognition, anxiety performances, anxiety sources, self-adjustment and coping suggestions in the open code are the sub categories of the main category. Based on this, the story of teachers’ occupational anxiety caused by the ‘double reduction’ policy is gradually clear: under the trigger of anxiety sources (causal conditions) such as the ‘double reduction’ policy narrowing the path of students’ knowledge acquisition, unreasonable teachers’ own work planning and parents’ inability to coordinate, primary and junior high school teachers gradually showed anxiety (phenomenon) such as doubting their own teaching ability, worrying about students’ learning effects and role conflicts due to the ‘double reduction’ policy. Furthermore, under the influence of the external environment such as unreasonable school management, lack of adequate humanistic care, implicit pressure exerted by teachers’ peers, families and society (context), teachers gradually chose to seek self-adjustment (action) through diet, sleep, mentality and other ways based on their different subjective perceptions of the ‘double reduction’ policy (intervening conditions). But ultimately, teachers’ anxiety could only be temporarily alleviated, and it would still recur later, and they were confused about how to deal with it effectively (consequence).

### 3.3. Selective coding

The selective coding is a process of finding the internal relationship between the main categories and systematically selecting the categories to find the core category. This study used selective coding to describe the relationship of each sequence, focusing on the story of ‘the causes of teacher’ occupational anxiety caused by the double reduction policy’. This part first explains the meaning of each category and its sub categories in detail.

#### 3.3.1. Teachers’ policy cognition

Teachers’ policy cognition referred to teachers’ understanding of the implementation effect of the ‘double reduction’ policy, mainly including positive effect and negative influence.

**Positive effect**. Teachers, based on their own education and teaching experience, believed that for teachers themselves, the introduction and implementation of the ‘double reduction’ policy would help teachers break through the old educational concepts and methods, as a whole could ‘force teachers to grow’ (T2, T11, T26, T38), and teachers would also be more active or conscious ‘to improve the teaching efficiency in the classroom’ (T1, T9, T25, T36). For students, under the ‘double reduction’, the more obvious changes were ‘the reduction of students’ homework’ (T7, T15, T17, T23), ‘the increase of students’ after-school time and rest time’ (T4, T6, T13, T30, T37, T41). At the same time, the overall school curriculum was also more abundant, and ‘students’ activities have become more’ (T24, T33).

**Negative influence**. Due to different schooling stages and disciplines, some teachers believed that the implementation of ‘double reduction’ had ‘seriously increased the burden on teachers’ (T2, T10, T14, T37), ‘increased the workload of teachers’ (T5, T19, T32), and ‘significantly extended the working hours’ (T1, T12, T35) when reducing the burden on students and parents. When it came to the lower grades of primary school and the first grade of junior high school, the ‘double reduction’ in reducing the pressure on students, on the contrary, ‘the test of students’ consciousness was very big’ (T14). Some teachers even thought that the relevant requirements of the ‘double reduction’ had affected the students in the first and second grades of primary school to lay a solid foundation to a certain extent. By the third grade, there would be a ‘big gap in academic level’ (T4, T8, T27) and ‘pseudo behavior’ (T4, T11, T29). In addition, although the number of after-school training institutions had decreased, parents’ attention to the quality of education and the objective impact of the pressure to enter higher education, such as ‘tutoring’ (T3, T8, T18, T25), ‘secretly coaching’ (T16, T21, T39), still existed, which had not really reduced the pressure on students.

Even though students may have to finish after-school services at five or six o’clock in school, parents will still choose tutors for their children, because some parents don’t care how much their children gain in school, they only care how much better their children are than others. Therefore, students just have less homework, but there is always pressure from exams and grades. It can be said that students’ original pressure has not decreased, and some even greater. (T3)

#### 3.3.2. Anxiety performances

Anxiety performances were specific states of teachers when they were anxious, which mainly included three aspects: self-development, students’ learning and role conflict.

**Self-development**. During the implementation of ‘double reduction’, teachers had to improve their own teaching quality, because ‘students’ performance was actually linked to the stability of teachers’ work’ (T1, T22), especially for teachers who were not authorized, they faced the risk of being dismissed at any time. The ‘double reduction’ policy not only alleviated students’ homework burden, but also put forward higher requirements for teachers’ teaching and homework design. Some teachers ‘feared that they could not do well and complete the task’ (T3, T20, T27, T44) and ‘doubted their teaching ability’ (T6, T16, T28). In addition, due to the impact of the COVID-19 and regional economic development, many teachers said that ‘the salaries for after-school services could not be paid’ (T1, T4, T17, T22), ‘teachers’ pay and income were not equal’ (T3, T9, T32, T40), and even for the moonlight clan, ‘it might be difficult to guarantee even basic life’ (T5, T13).

**Student learning**. Whether the teaching effect was good or not was also one of the teachers’ occupational anxiety caused by the ‘double reduction’ policy. Therefore, the ‘uncertainty and unknown’ (T1, T2, T7, T10, T45) of students’ learning had been bothering in-service teachers.

With the implementation of the ‘double reduction’ policy, I am not particularly sure whether students’ knowledge is solid or not, and whether it will affect their knowledge acquisition in the future. That is to say, I cannot guarantee that they will be able to learn well under this background. Therefore, on the whole, I am not very sure about students’ learning effectiveness. (T2)

**Role conflict**. The role of teachers was diverse, and the implementation of ‘double reduction’ had led to many role conflicts for teachers to some extent. As far as students were concerned, they were ‘educators and helpers’. As far as families were concerned, they were children and parents. Some teachers found that many parents tried to find better resources, and they would also have the idea of ‘finding ways to narrow the gap between their children and other children’ (T8, T23). At the same time, due to the increase of after-school services, homework design and other tasks, many teachers said that ‘they had no time to accompany their children and feel sorry for them’ (T13, T19, T35). In addition, teachers also played the role of colleagues or peers. Some believed that the implementation of ‘double reduction’ made teachers ‘more involved and competitive’ (T31, T39), and all competed for time to train students at different levels. Between family and school, teachers also played the role of ‘communicators’, but in reality, many in-service primary and junior high school teachers faced serious problems, because the continuous existence of family-school conflicts had led to teachers’ anxiety. (T4, T20, T26).

#### 3.3.3. Anxiety sources

Anxiety sources referred to the root causes of teachers’ occupational anxiety. The interview and analysis results showed that teachers’ anxiety mainly came from the ‘double reduction’ policy, teachers’ individual factors and parents’ weak coordination.

**The ‘double reduction’ policy**. During the implementation of the policy, some in-service teachers found that the ‘double reduction’ policy triggered teachers’ occupational anxiety.

I think my anxiety is also related to ‘double reduction’, because students really have many ways to acquire knowledge, but now their only approach is to study at school. Therefore, teachers are bound to be anxious under greater pressure. (T43)

**Teachers’ individual factors**. Many in-service teachers said that on the one hand, occupational anxiety was caused by their own ‘sense of responsibility’ (T1, T2, T5, T11, T44), which wanted to teach students well and let them learn more under the ‘double reduction’. Especially for junior high schools, the reality of ‘general-vocational separation’ was the trend. Teachers who were in junior high school wanted to make students go to general high schools as much as possible (T15, T24, T36), so teachers would bear more pressure and generate anxiety. On the other hand, ‘double reduction’ put forward higher requirements for teachers. Some teachers adapted slowly, which led to difficulty in coordinating time and task planning, ‘imbalance between life and work’, ‘chaos when busy’ and other situations were appeared. (T6, T7, T9, T12, T34, T42).

**Parents’ weak coordination**. As a key participant in the education process, many parents believed that the implementation of ‘double reduction’ was a unilateral task of teachers. They blindly ‘exerted pressure on teachers’ and ‘did not cooperate with teachers’ (T3, T11, T19, T26, T38), which undoubtedly brought a lot of inconvenience to teachers’ work, and was one of the root cause of teachers’ occupational anxiety.

#### 3.3.4. Self-adjustment

Self-adjustment was a personal adjustment mode that teachers chose when facing anxiety, mainly involving internal adjustment and external support.

**Internal adjustment.** Through the interview, it was found that the in-service primary and junior high school teachers mentioned the importance of self psychological adjustment, and believed that a good attitude could transform anxiety into work motivation to a certain extent (T7, T10, T17, T31, T45). In addition, some teachers also chose to adjust themselves through ‘eating delicious food’ (T1, T12, T19, T27), ‘supplementing sleep’ (T6, T33) and ‘reasonably planning teaching work and time’ (T18, T25, T34).

**External support.** In addition to the necessary internal self-adjustment, in-service primary and junior high school teachers said that external support was very important. For teachers with children, having someone at home to help with childcare could reduce the pressure of busy teaching work (T14, T15, T33, T40). At the same time, under the ‘double reduction’ policy, schools could provide necessary humanistic care, create a good working atmosphere and a united colleague relationship, which were also effective ways to alleviate teachers’ occupational anxiety. (T8, T15, T21, T32).

#### 3.3.5. Coping suggestions

Coping suggestions referred to the specific strategies proposed by teachers to alleviate anxiety based on their own experience, including subjective level and objective condition.

**Subjective level**. When talking about solving anxiety problems, in-service primary and junior high school teachers thought that teachers needed to learn to adapt to the ‘double reduction’ policy and actively support the implementation of the policy (T5, T7, T18, T23), and needed to ‘adjust their mentality’ in time (T1, T3, T10, T13, T25, T28, T36, T45) to improve their psychological building ability. At the same time, teachers also needed to constantly improve their work efficiency (T2, T9, T14, T34), adhere to active learning (T3, T10, T21, T37), and learn to speak out or reduce responsibility appropriately (T22, T40). Anxiety could be gradually alleviated only if teachers’ professional quality continued to improve.

**Objective condition**. Based on the consideration of the problems such as unequal pay and return, heavy burden and so on, many teachers said that the ‘double reduction’ policy needed to be further improved and ‘implemented from top to bottom’ (T4, T9, T11, T16, T35, T42). In addition, in-service teachers also believed that society should give more tolerance to teachers, schools should create a harmonious and democratic environment, and teachers also needed to actively cooperate (T5, T7, T8, T12, T17, T20, T26, T39), which would help teachers break through the dilemma of occupational anxiety.

To sum up, the implementation of the ‘double reduction’ policy was affected by many factors, which led to the gradual exposure of social, school, family and other contradictions in the education system, thus affecting the orderly teaching of in-service primary and junior high school teachers and causing their anxiety. As a result, the core category of research emerged, that is, the implementation of the ‘double reduction’ policy was incomplete matching the actual education ecology. Based on this, the researchers further clarified the attributes and dimensions of the core category (see Table 4).

Through an in-depth analysis of core category, this study found that the implementation of the ‘double reduction’ policy and actual education ecology were affected by many factors, and there was a complex relationship (see Figure 4).

### 3.4. A theoretical model of the formation mechanism of teachers’ occupational anxiety caused by the ‘double reduction’ policy

In order to deeply explain the generation mechanism of teachers’ occupational anxiety, this study constructed a theoretical model of the formation mechanism of teachers’ occupational anxiety with the core of ‘the implementation of the double reduction policy was incomplete match actual education ecology’ (see Figure 5). Focusing on teachers’ occupational anxiety performances, on the one hand, it stemmed from the fact that the implementation of the ‘double reduction’ policy did not fully match actual education ecology, that was, the implementation of the ‘double reduction’ policy at multiple levels, such as reducing students’ homework burden, managing after-school training, and improving teachers’ professional quality, was affected by many factors, such as the economy, family-school-community, the COVID-19, which led to deviation between the actual educational ecology and the preset, causing teachers’ occupational anxiety. At the same time, teachers in primary and junior high schools had different understanding of the ‘double reduction’ policy, and some teachers were timid and submissive, which, to a certain extent, had also become the cause of their own anxiety.

However, focusing on teachers’ occupational anxiety itself, it could mainly be summarized as three manifestations: perplexed by the uncertainty of their own development, panic about the unknown learning effect of students, and helplessness in coordinating role conflicts. Specifically, in terms of their own development, teachers felt more confused about their career development due to the instability of their posts, insufficient teaching ability and unequal pay and return. For students’ learning, teachers always worried about whether students could achieve effective learning under the ‘double subtraction’ policy. At the same time, teachers’ occupational anxiety was also aggravated by the conflicts of multiple roles such as ‘educators’, ‘peers’ and ‘communicators’. In fact, in the face of the objective existence of anxiety, in-service primary and junior high school teachers had always insisted on making efforts to self adjust, but the effect was often poor. Therefore, they sincerely hoped that external help could be provided to better help them alleviate anxiety. However, in reality, external support was obviously insufficient. It was obvious that the occupational anxiety of teachers caused by the ‘double reduction’ policy was the result of multiple factors, among which, those factor influence and restrict each other. In a word, although teachers were affected by the mismatch between their own personality characteristics and external factors, they could relieve their anxiety in a timely manner through active self-adjustment, which was actually conducive to further promoting the smooth implementation of the ‘double reduction’ policy and the construction of a good educational ecology.

## 4. Discussion and conclusion

### 4.1. Summary and discussion

With the help of the grounded theory, this study analyzed the formation mechanism of teachers’ occupational anxiety caused by the ‘double reduction’ policy. The main findings of this study would be further discussed in depth in comparison with existing relevant studies.

First of all, this study found that in-service primary and junior high school teachers had different understanding of the ‘double reduction’ policy, which affected the development of their anxiety state to a certain extent. Faced with the rapid and complex educational reform, teachers were easy to have occupational anxiety. This anxiety was not only caused by the objective factors of the uncertainty and fuzziness of educational reform itself, but also mainly depended on the subjective understanding of different individual teachers on educational reform. Therefore, teachers’ beliefs and state always played an important role in educational reform (Ham and Dekkers, 2019). In essence, ‘double reduction’ was a process of educational reform. In this context, teachers’ occupational anxiety affected the smooth implementation of the policy, which was mutually confirmed by existing research. At the same time, through the analysis of the interview text, when asked, ‘If you were given the choice now, would you still choose the profession of teachers? Please explain the reason.’ Some teachers replied that they were ‘uncertain’ and worried about their teaching ability and job stability. This showed that the resilience of teachers was affected by many internal and external factors (Phillips, 2021), that was, many in-service primary and junior high school teachers had shaken their original teaching intentions to a certain extent, and even some teachers had doubts and resistance to the ‘double reduction’ policy.

Secondly, during the interview, this study also found that there was widespread ‘role anxiety’ among in-service primary and junior high school teachers. Especially for in-service primary school teachers, in addition to being teachers, they also acted as ‘nurseries’, ‘security officers’ in afternoon care and after-school care, ‘activity planners’ before carrying out activities, ‘grid members’ who collected various health codes or itinerary codes during the COVID-19, and ‘accountant’ to assist school leaders in accounting statistics. Outside the school, teachers also played the role of ‘parents’ and ‘children’. As a result, in the face of the heavy teaching tasks under the ‘double reduction’ policy and the multiple role conflicts, teachers’ occupational anxiety arose spontaneously, which became the dominant force hidden behind the teachers’ role and constituted the psychological motivation for teachers to complain, resist and escape. At the same time, through interviews, this study verified the view that ‘teachers had role conflicts between work and family’ (Cinamon and Rich, 2005; Li et al., 2021) from an empirical perspective. On the other hand, teachers’ multiple roles in the school were all symbols of non-educational teaching tasks that teachers undertake. These matters that had no close relationship with teachers’ own work led to teachers’ lack of enough time and energy to devote to their own work, thus resulting in heavier work pressure. Therefore, teachers’ occupational anxiety should not be ignored.

Finally, through in-depth interviews, many in-service primary and junior high school teachers reported that their contributions and rewards were completely unequal, and they believed that their sense of teaching achievement and work enthusiasm had been eroded year by year. Especially in after-school services, some teachers said that they had worked hard to design courses and implement activities, and finally they might only receive a few yuan for each class. For young teachers, some said that it was difficult to maintain their basic salary and they often needed help from their parents. This was the same as the view put forward in the existing research that teachers’ pay and return were unbalanced in their work (Unterbrink et al., 2007; Hinz et al., 2016; Ren et al., 2019; Guo et al., 2022).

In addition to many similarities with previous studies, this study also highlighted some new factors. For example, different from previous studies on teachers’ occupational anxiety, this study was based on the new era background, that was, to explore teachers’ occupational anxiety caused by the ‘double reduction’ policy, the perspectives or information of ‘students’ pseudo learning behavior’, ‘teachers’ anxiety about after-school services’ and ‘worried about students’ narrow access to study’ were innovative. At the same time, focused on teachers themselves, although teachers generally had anxiety caused by the ‘double reduction’ policy, they were still driven by their educational conscience and sense of responsibility. On the one hand, they always insisted on passing on what they knew to students in a positive way, on the other hand, they actively adjusted themselves through diet, sleep and other ways to digest their negative emotions promptly. In addition, through in-depth interviews, this study also obtained teachers’ heartfelt coping suggestions on alleviating anxiety, which to some extent provided strong support for the follow-up in-depth research.

### 4.2. Limitations

Of course, limited by the research data and materials, we had not yet used quantitative research methods to verify the conclusions of this study. We will conduct statistical tests on the conclusions in the next study. At the same time, as the ‘double reduction’ is still in the process of dynamic development, it is worth continuing to think about whether the problem of incomplete match between the policy implementation and the actual education ecology will ease with the development of time. Although this research has constructed the formation mechanism of teachers’ occupational anxiety caused by the ‘double reduction’ policy, it is still open to the possibility of its development, which is also the continuous motivation for us to continue our research.

## 5. Conclusion

In order to deeply explore the problem of teachers’ occupational anxiety caused by the ‘double reduction’ policy, this study conducted in-depth interviews and data coding analysis on 45 in-service primary and junior high school teachers using the method of grounded theory. The study found that during the implementation of the ‘double reduction’ policy, teachers had occupational anxiety, which had a negative impact on their work and life. Although this finding has verified the relevant conclusions of existing studies to some extent (see Section 4.1 for details), the main contributions and highlights of this study, in addition to clarifying the objective existence of the phenomenon, have also built a theoretical model of the formation mechanism of teacher’ professional anxiety caused by the ‘double reduction’ policy with the help of grounded theoretical methods. Among them, teachers’ occupational anxiety is mainly manifested in their concern about their uncertain development, students’ learning effectiveness and multiple role conflicts, which is not only the result of the incomplete match between the implementation of the ‘double reduction’ policy and the actual education ecology, but also affected by teachers’ personality characteristics. In addition, this study has also produced some new insights, that is, teachers are not passively exposed to occupational anxiety, but can actively alleviate anxiety through self-adjustment. At the same time, teachers also expressed their own aspirations, and further put forward suggestions on the subjective level, including actively adapting to policies, adjusting mentality, improving their own work efficiency, as well as on the objective level, such as improving policies and building collaborative relationships. However, it is far from enough to rely on the efforts of teachers alone, because the improvement of the ‘double reduction’ policy and the implementation of teachers’ after-school service pay all need the help of the relevant national departments. To create a harmonious, democratic and cooperative working atmosphere in the school, reasonably arrange teachers’ work tasks and other urgent needs the effective management of the school departments at all levels, and the comprehensive development of students also requires family education to bear the natural responsibility. Therefore, only when all parts and members perform their own duties, can the ‘double reduction’ policy be truly implemented, teachers’ anxiety will gradually ease, and then return to the true role of ‘teaching’.

## Data availability statement

The original contributions presented in the study are included in the article/supplementary material, further inquiries can be directed to the corresponding author.

## Ethics statement

Ethical review and approval was not required for the study on human participants in accordance with the local legislation and institutional requirements. Written informed consent for participation was not required for this study in accordance with the national legislation and the institutional requirements.

## Author contributions

WY, LY, and YY contributed to the ideas of educational research. WY contributed to the contacting participant and collection of data. LY, YY, and WY contributed to the data analysis, design of research methods, and tables. WY, LY, and YY participated in writing and revision. All authors contributed to the article and approved the submitted version.

## Funding

This research was funded by the Major Project ‘National Research on Environment and Sustainable Development Education’ Funded by the Ministry of Education of the People’s Republic of China in the Post-funded of Philosophy and Social Science Research in 2022 (Grant Number 22JHQ018).

## Conflict of interest

The authors declare that the research was conducted in the absence of any commercial or financial relationships that could be construed as a potential conflict of interest.

## Publisher’s note

All claims expressed in this article are solely those of the authors and do not necessarily represent those of their affiliated organizations, or those of the publisher, the editors and the reviewers. Any product that may be evaluated in this article, or claim that may be made by its manufacturer, is not guaranteed or endorsed by the publisher.
